# Histone Demethylase LSD1 Regulates Kidney Cancer Progression by Modulating Androgen Receptor Activity

**DOI:** 10.3390/ijms21176089

**Published:** 2020-08-24

**Authors:** Kyoung-Hwa Lee, Byung-Chan Kim, Seung-Hwan Jeong, Chang Wook Jeong, Ja Hyeon Ku, Cheol Kwak, Hyeon Hoe Kim

**Affiliations:** 1 Department of Urology, Seoul National University Hospital, Seoul 03080, Korea; Lee12042@snu.ac.kr (K.-H.L.); dalkyal12@gmail.com (B.-C.K.); drboss@snu.ac.kr (C.W.J.); randyku@hanmail.net (J.H.K.); 2 Graduate School of Medical Science and Engineering, Korea Advanced Institute of Science and Technology (KAIST), Daejeon 34052, Korea; 11shjeong@kaist.ac.kr; 3 Department of Urology, Seoul National University College of Medicine, Seoul 03080, Korea

**Keywords:** androgen receptor, histone methylation, LSD1, kidney cancer, enzalutamide

## Abstract

Kidney cancer is one of the most difficult cancers to treat by targeted and radiation therapy. Therefore, identifying key regulators in this cancer is especially important for finding new drugs. We focused on androgen receptor (AR) regulation by its epigenetic co-regulator lysine-specific histone demethylase 1 (LSD1) in kidney cancer development. LSD1 knock-down in kidney cancer cells decreased expression of AR target genes. Moreover, the binding of AR to target gene promoters was reduced and histone methylation status was changed in LSD1 knock-down kidney cancer cells. LSD1 knock-down also slowed growth and decreased the migration ability of kidney cancer cells. We found that pargyline, known as a LSD1 inhibitor, can reduce AR activity in kidney cancer cells. The treatment of kidney cancer cells with pargyline delayed growth and repressed epithelial–mesenchymal transition (EMT) markers. These effects were additively enhanced by co-treatment with the AR inhibitor enzalutamide. Down-regulation of LSD1 in renal cancer cells (RCC) attenuated in vivo tumor growth in a xenograft mouse model. These results provide evidence that LSD1 can regulate kidney cancer cell growth *via* epigenetic control of AR transcription factors and that LSD1 inhibitors may be good candidate drugs for treating kidney cancer.

## 1. Introduction

Kidney cancer is one of the most lethal types of cancer, accounting for ~3% of malignancies in the USA in 2018 [1]. The mechanism underlying the pathogenesis of kidney cancer is poorly understood and its detection remains difficult. Moreover, the tumors are usually resistant to chemotherapy and radiation therapy, and targeted therapy agents, such as sunitinib, sorafenib, bevacizumab, and temsirolimus, have also shown very limited results. Therefore, the identification of new diagnostic markers and therapeutic targets for kidney cancer is imperative.

The androgen receptor (AR) is a major transcription factor responding to androgenic hormones in males. However, androgens are also circulating at physiologically relevant levels in women and are thus thought to have different biological roles besides the male sex hormone function. The AR plays a role in cell proliferation and migration in many types of cancers, including colon [2], breast [3,4], stomach [5], and bladder cancer [6,7,8,9], in addition to prostate cancer. Renal cell carcinoma (RCC) is also considered to be affected by AR due to the reported gender difference in its incidence, which is higher in males than females [1]. In contrast with its function in other cancers, the expression of AR in RCC is negatively correlated with cancer development and survival rate [10,11]; however, some reports show that the AR level influences the initiation and metastatic route of RCC [12,13,14]. These conflicting results might suggest that a change in AR activity, rather than expression level, is more important in kidney cancer. Therefore, it is worth elucidating the detailed mechanism of AR activity regulation in RCC. To analyse AR function in kidney cancer, we selected Caki-2 cells among several RCC cell lines because there are reports of AR expression in this cell line [15].

Among the mechanisms known to regulate AR transcription factors, epigenetic regulation, including DNA methylation and histone acetylation and methylation, plays a key role. Several transcriptional co-factors were reported as regulators of AR activity. P300/CBP-associated factor (PCAF), p300/CBP, heat shock protein 90 (HSP90), Tip60, and SWI/SNF associate with the transcription complex [16,17,18,19], and many histone methyl-transferases and demethylases control AR-responsive gene regulation. Among the epigenetic regulators of AR, lysine-specific histone demethylase 1 (LSD1, also known as KDM1A) was discovered first, and its mechanism has been well studied in prostate cancer [20]. LSD1 can demethylate histone H3K4 and H3K9 depending on its interacting partners [21,22]. For example, LSD1 activates its partner sex hormone transcription factors, including oestrogen receptor (ER) and AR, by removing the methyl group repressive mark H3K9 [4,20,23,24].

LSD1 is a flavin-dependent demethylase and its catalytic mechanism involves the oxidation of flavin adenine dinucleotide (FAD) and consumption of O_2_. Its close homology to monoamine oxidase (MAO) explains the inhibitory potency of several MAO inhibitors towards LSD1. These include FDA-approved drugs for the treatment of depression or hypertension, such as tranylcypromine (PCPA), pargyline, and clorgyline [25]. These inhibitors show great potency for LSD1 in the low nM range and are very promising in preclinical cancer investigations [26,27]. In this report, we tested pargyline as an LSD1 inhibitor to explore its potential as an anti-kidney cancer drug.

Here, we focused on the role of LSD1 in the regulation of AR activity in kidney cancer growth and metastasis, and explored the possible use of LSD1 chemical inhibitors for kidney cancer treatment. Furthermore, the additive effect of LSD1 inhibitor together with the existing AR inhibitor enzalutamide was evaluated.

## 2. Results

### 2.1. LSD1 Regulate AR Activity and Cell Proliferation in RCC

**Figure 1:**
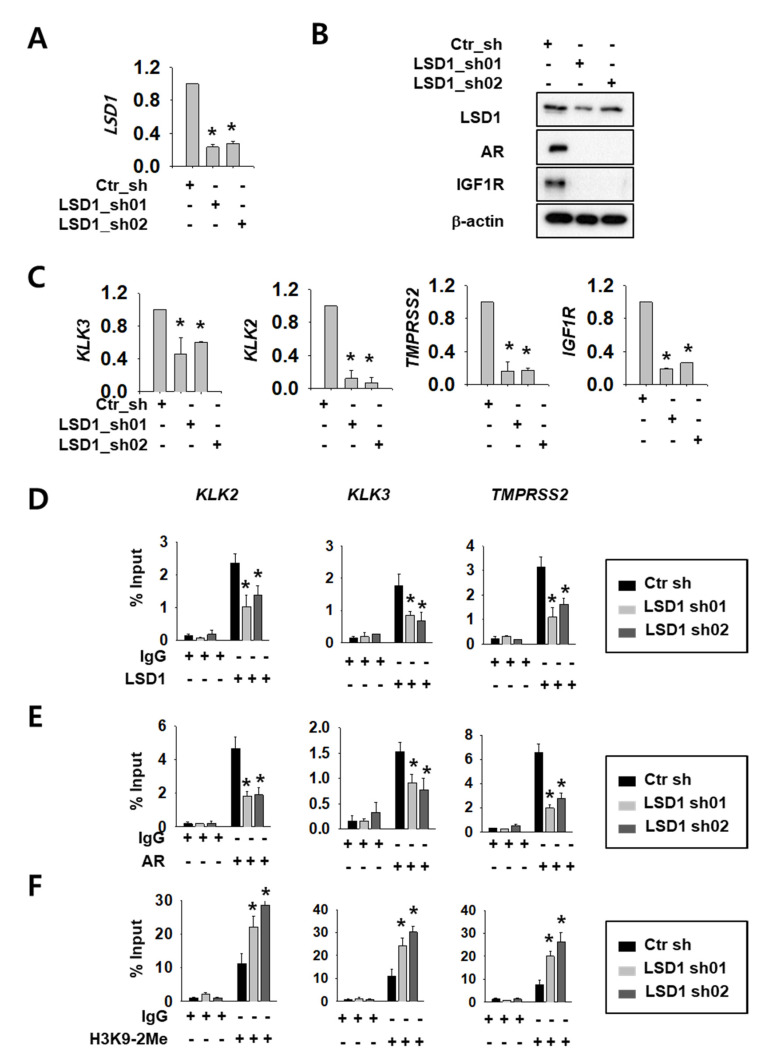
Lysine-specific histone demethylase 1 (LSD1) knock-down reduces androgen receptor (AR) activity by changing binding and histone methylation on target promoters in kidney cancer cells. (**A**) LSD1 messenger RNA (mRNA) knock-down was confirmed by reverse transcription quantitative polymerase chain reaction (RT-qPCR) in Caki-2 cells. Bars represent the means ± SD of three independent experiments, and * denotes *p* < 0.05 (Student’s *t*-test) versus the control shRNA (ctr_sh) group. (**B**) LSD1 knock-down and AR target protein expression were analysed with Western blotting. Extracts from each cell line were analysed with indicated antibodies. (**C**) The mRNA level of AR target genes was analysed by RT-qPCR in LSD1 knock-down Caki-2 cells. Bars represent the means ± SD of three independent experiments, and * denotes *p* < 0.05 (Student’s *t*-test) versus the ctr_sh group. (**D**–**F**) Fragmented chromatin complexes from each cell line were immunoprecipitated with anti-LSD1 (**D**), anti-AR (**E**), or anti-H3K9-2Me (**F**) antibodies. The immunoprecipitated DNAs were analyzed by PCR using primers based on gene promoter sequence, as indicated above. Bars represent the means ± SD of three independent experiments, and * denotes *p* < 0.05 (Student’s *t*-test) versus the ctr_sh group.

We selected Caki-2 cells among several RCC cell lines because there are reports of AR expression [16]. We confirmed AR protein expression (Supplemental Figure S1A) and cell-growth reduction upon AR knock-down in these cells (Supplemental Figure S1B,C). To investigate LSD1 function as an AR regulator, we expressed control or LSD1 short hairpin RNA (shRNA)s using lentiviral system in Caki-2 kidney cancer cells. The knock-down efficiency was confirmed by reverse transcription quantitative polymerase chain reaction (RT-qPCR) methods (Figure 1A) and Western blotting (Figure 1B). Next, we assessed whether LSD1 modulation affects AR activity in kidney cancer cells. To achieve this aim, we tested expression of AR target genes at the messenger RNA (mRNA) level, as well as protein levels. LSD1 knock-down cells showed reduced expression of AR downstream genes including AR itself and insulin-like growth factor 1 receptor (IGF1R) at the protein level (Figure 1B). AR downstream genes, including kallikrein related peptidase (KLK) 3, KLK2, transmembrane protease serine subtype 2 (TMPRSS2), and IGF1R, were also significantly reduced at the mRNA level upon LSD1 knock-down (Figure 1C). To verify the molecular mechanism of LSD1 on AR target promoters, we performed chromatin immunoprecipitation using anti-LSD1 and anti-AR antibodies. We found that LSD1 binds to AR target genes including KLK2, KLK3, and TMPRSS2 in kidney cancer cells, and confirmed that the binding decreased in LSD1 shRNA-expressing cells (Figure 1D). AR binding to these promoters also decreased in LSD1 knock-down cells (Figure 1E). As LSD1 is known to demethylate H3K9 while interacting with AR [20], we performed immunoprecipitation with anti-histone H3K9 di-methyl antibody in LSD1 knock-down cells. We found that the PCR signals for AR target gene promoters from the precipitates increased in LSD1 knock-down cells (Figure 1F). These data show that LSD1 interacts with AR in its target genes and activates AR transcription factors *via* demethylating histone H3K9.

### 2.2. LSD1 Down-Regulation Reduces Kidney Cancer Cell Proliferation and Increases Anti-Cancer Drug Sensitivity

**Figure 2:**
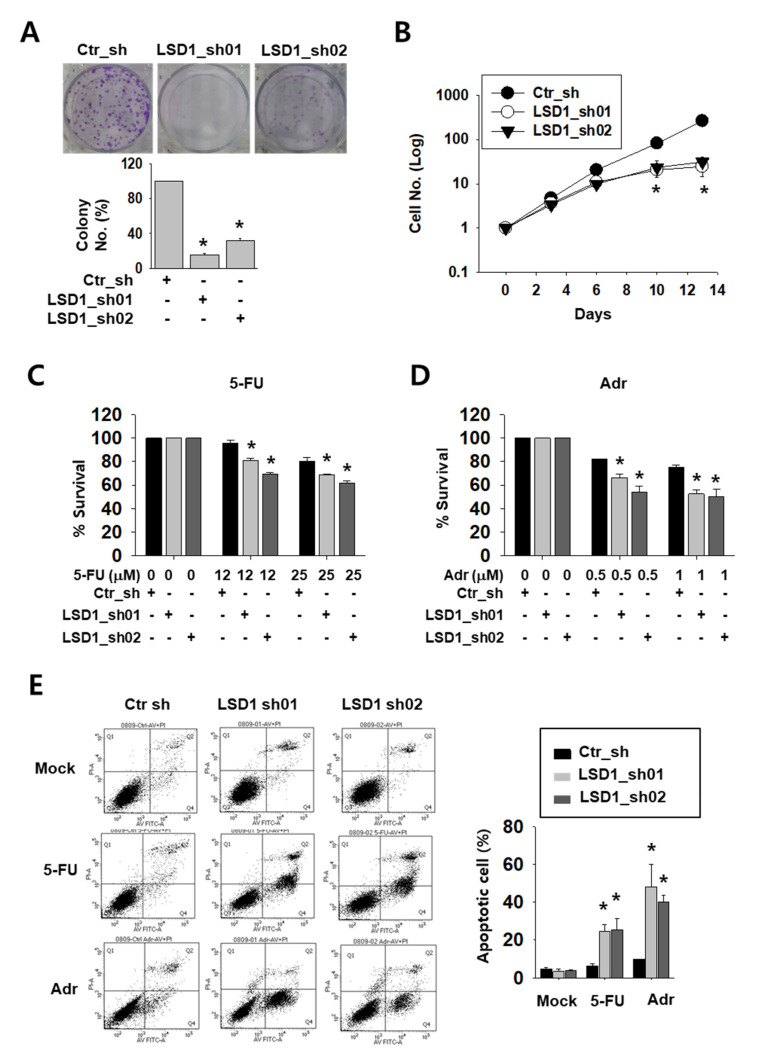
LSD1 knock-down reduces cell proliferation and increases the effect of anti-cancer drugs on growth reduction in kidney cancer cells. (**A**) Crystal violet staining for colonies from the same number of indicated short hairpin RNA (shRNA)-expressing Caki-2 cells. The average number of colonies was counted and is shown in the lower graph. Bars represent the means ± SD of three independent experiments, and * denotes *p* < 0.05 (Student’s *t*-test) versus the ctr_sh group. (**B**) The growth curve of control and LSD1 shRNA-expressing Caki-2 cells. Cells were counted on the indicated day and the cell numbers were evaluated. The results are shown as the mean ± SD of three independent experiments, and * denotes *p* < 0.05 (Student’s *t*-test) versus the ctr_sh group. (**C**,**D**) Cell viability of control or LSD1 knock-down Caki-2 cells after treatment with 5-fluoro-uracil (5-FU) (**C**) or adriamycin (**D**) at indicated concentrations. Relative optical density values were measured using EZ-cytox solution after two days of drug treatment. Bars represent the means ± SD of three independent experiments, and * denotes *p* < 0.05 (Student’s *t*-test) versus the ctr_sh group. (**E**) Flow cytometry analysis of annexin V and propidium iodide (PI) staining following 5-FU (25 µM) or adriamycin (1 µM) treatment for three days in LSD1 knock-down cells. The percentage of apoptotic cells (annexin V-positive) is graphed. Bars represent the means ± SD of three independent experiments, and * denotes *p* < 0.05 (Student’s *t*-test) versus the ctr_sh group.

To test the possible effect of LSD1 on kidney cancer cell growth, we tested the colony formation and counted cell growth of Caki-2 cells. LSD1 knock-down cells formed less and smaller colonies than control cells (Figure 2A). Moreover, while control cells showed an exponential growth curve, LSD1 knock-down cells showed a significantly slower growth curve over a period of 14 days (Figure 2B). Next, we treated knock-down cells with anti-cancer drugs, including DNA-damaging reagent 5-fluoro-uracil (5-FU) or adriamycin (Adr), to test their sensitivity. As shown by the optical density measurement, LSD1 knock-down cells showed a higher sensitivity to these drugs than control cells (Figure 2C,D). We then analysed apoptotic cells in the anti-cancer drug-treated cells. Following drug treatment, the number of annexin V-stained apoptotic cells were measured using fluorescence-activated cell sorting (FACS). After 5-FU or Adr treatment, the percentage of total apoptotic cells was dramatically increased in the LSD1 knock-down cells (Figure 2E). These data confirmed that LSD1 controls cell proliferation and showed that LSD1 repression can increase anti-cancer drug sensitivity in kidney cancer cells.

### 2.3. LSD1 Reduction Slowed Kidney Cancer Cell Migration and Decreased Epithelial–Mesenchymal Transition (EMT) Marker Expression

**Figure 3:**
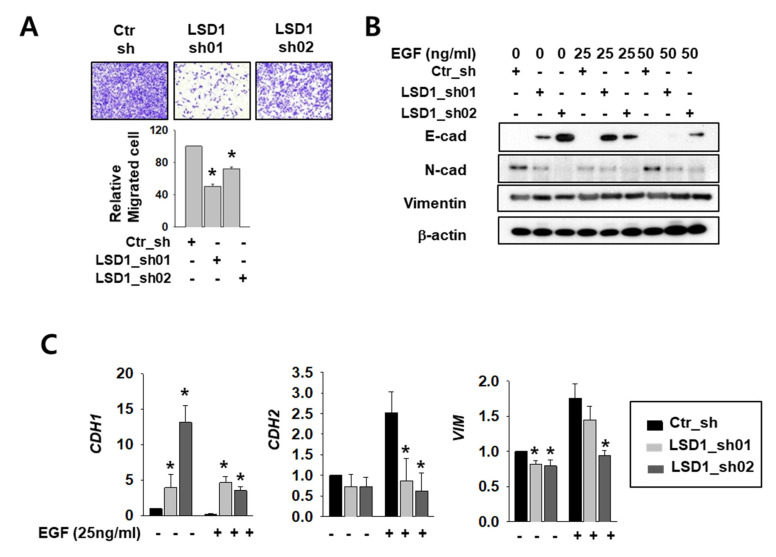
LSD1 knock-down reduces cell mobility and epithelial–mesenchymal transition (EMT)-related gene expression in kidney cancer cell lines. (**A**) The transwell infiltration assay of the same number of indicated shRNA-expressing Caki-2 cells was observed after two days of incubation. Cells that had migrated to the underside of the filters were fixed and photographed. Graph presents the invasion rate as the mean percentage of control. Error bars represent the mean ± SD of three independent experiments and * denotes *p* < 0.05 (Student’s *t*-test) versus ctr_sh group. (**B**) Western blotting of indicated EMT markers after 24 h treatment with indicated concentrations of epidermal growth factor (EGF) in control or LSD1 shRNA-expressing cells. (**C**) Quantitative PCR was used to examine the transcriptional level of the EMT marker genes in Caki-2 cells expressing control or LSD1 shRNA after two days of EGF treatment. Error bars represent the mean ± SD of three independent experiments and * denotes *p* < 0.05 (Student’s *t*-test) versus ctr_sh group.

The AR functions in cell migration and cancer metastasis by regulating its major target genes E-cadherin (CDH1) and vimentin (VIM) [4,28]. To clarify the role of LSD1 in RCC metastasis, we measured differences in cell infiltration between control and LSD1 knock-down Caki-2 cells. The invasion assay showed that the LSD1-suppressed cells infiltrated less than the control cells (Figure 3A). To induce epithelial–mesenchymal transition (EMT), we treated control and LSD1 knock-down cells with epidermal growth factor (EGF). The protein level of mesenchymal markers N-cadherin and vimentin decreased while epithelial marker E-cadherin protein increased in LSD1 knock-down cells before and after EGF treatment (Figure 3B). Again, the mRNA level of CDH1 was dramatically increased while that of N-cadherin (CDH2) and VIM decreased in LSD1-suppressed cells (Figure 3C). These data confirmed that LSD1 knock-down can suppress EMT in kidney cancer cells. Next, we treated control and LSD1 knock-down Caki-2 cells with AR antagonist enzalutamide. Enzalutamide treatment increases epithelial markers and decreases mesenchymal markers. Again, we found that CDH1 was up-regulated and CDH2 and VIM were down-regulated in LSD1 knock-down cells before and after enzalutamide treatment (Supplemental Figure S2). Taken together, LSD1 knock-down together with enzalutamide further suppresses EMT in kidney cancer cells.

### 2.4. Growth Attenuation and Apoptosis Induction After Enzalutamide Treatment are Additively Increased by LSD1 Inhibitor in Kidney Cancer Cells

**Figure 4:**
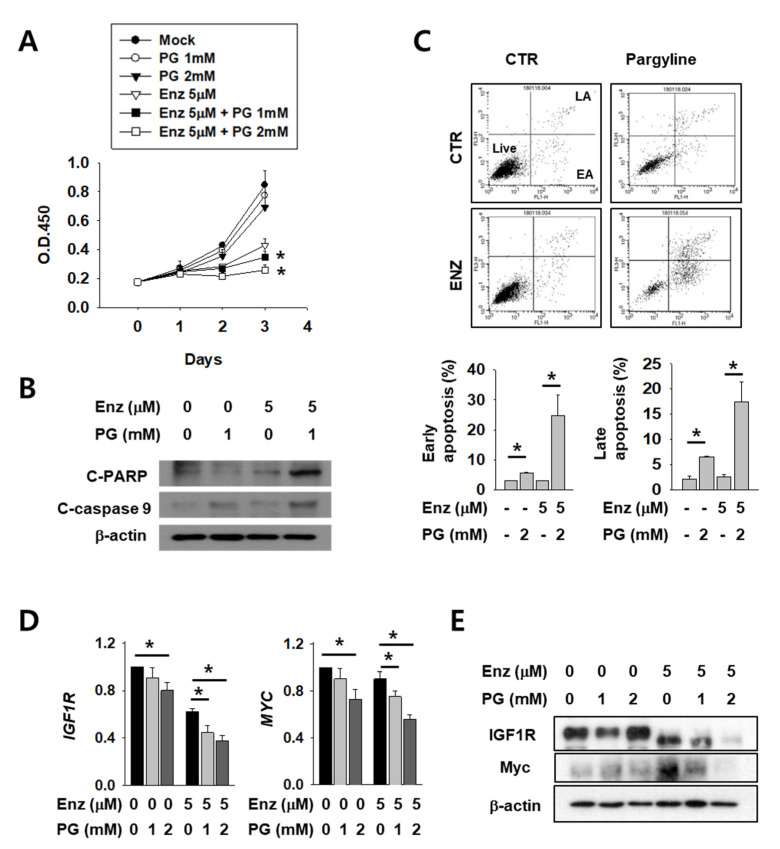
LSD1 inhibitor pargyline (PG) treatment attenuates kidney cancer growth and induces cell death additively with enzalutamide. (**A**) Caki-2 cells were treated with enzalutamide and/or the indicated concentrations of PG and the optical density values of viable cells were determined using EZ-Cytox solution. Bars represent the means ± SD of three independent experiments, and * denotes *p* < 0.05 (Student’s *t*-test) versus enzalutamide-only treated group. (**B**) After two days of treatment with the indicated drugs, the total cell lysates were collected and immunoblotted with the indicated antibodies. (**C**) Flow cytometry analysis of annexin V and propidium iodide (PI) staining of apoptotic cells following treatment of Caki-2 cells with the indicated drug for three days. The percentage of early apoptosis (EA) and late apoptosis (LA) cells are calculated and graphed below. Error bars represent the mean ± SD of three independent experiments and * denotes *p* < 0.05 (Student’s *t*-test) between the two indicated groups. (**D**) The mRNA levels of indicated genes in Caki-2 cells treated with enzalutamide and/or pargyline were analyzed by RT-qPCR. Error bars represent the mean ± SD of three independent experiments and * denotes *p* < 0.05 (Student’s *t*-test) between the two indicated groups. (**E**) Western blots of whole-cell lysates after 24 h of treatment with the indicated drugs and concentrations. Whole-cell lysates were immunoblotted with indicated antibodies.

Next, we tested the possible anti-cancer effect of LSD1 inhibitors on kidney cancer cells. AR antagonist enzalutamide is generally used for prostate cancer and is now under clinical trials for kidney cancer patients. As these two drugs act on AR in different stages and may have different mechanisms, we expected that co-administration of both would have an additive effect. To verify this hypothesis, we tested co-treatment with LSD1 inhibitor pargyline (PG) and enzalutamide. Enzalutamide alone successfully inhibited kidney cancer cell growth (Figure 4A). Interestingly, co-treatment with both drugs further decreased cell growth after 2–3 days of incubation. The levels of apoptosis markers, including cleaved poly (ADP-ribose) polymerase (PARP) and cleaved caspase-9, were significantly higher after co-treatment than with either enzalutamide or PG alone (Figure 4B). Subsequently, cell death was measured after the drug treatment, using FACS analysis. Both the early and late apoptotic cell populations were significantly increased in double-treated cells (Figure 4C). Next, we analysed AR activity by quantifying levels of its target genes IGF1R and MYC in enzalutamide and/or PG-treated kidney cancer cells. The IGF1R and MYC mRNAs were down-regulated after enzalutamide treatment alone, and were further reduced in a dose-dependent manner after PG co-treatment (Figure 4D). The protein levels of IGF1R and MYC also decreased further after PG co-treatment (Figure 4E). These results show that pargyline effectively inhibits AR, and AR inhibition by two different drugs, enzalutamide and pargyline, additively reduced kidney cancer cell growth and induced apoptosis.

### 2.5. The Effect of Enzalutamide on EMT Marker Reduction is Enhanced by PG Co-Treatment in Kidney Cancer Cells

**Figure 5:**
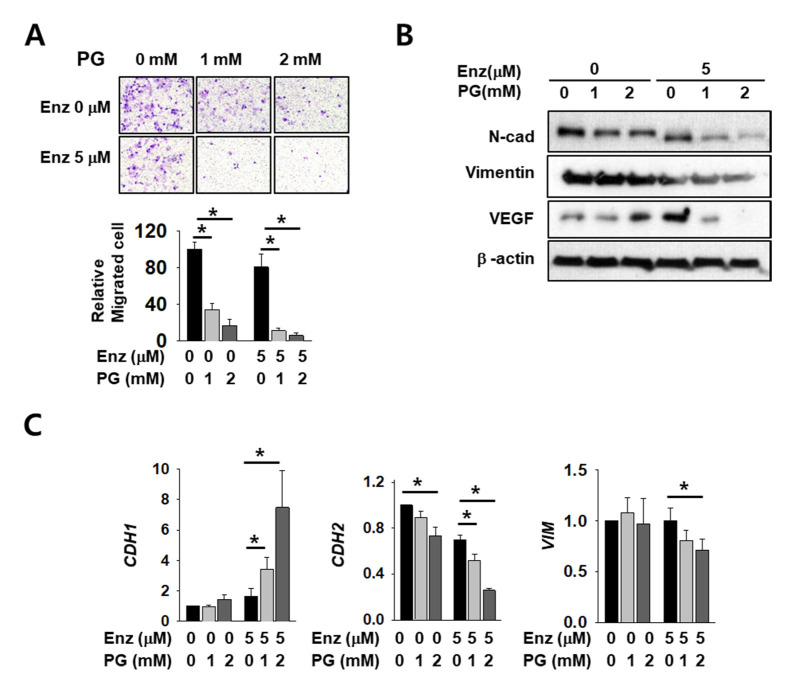
Pargyline treatment attenuates kidney cancer cell migration and decreases EMT marker expression. (**A**) The transwell infiltration assay of the same number of Caki-2 cells treated with indicated drugs observed after two days of incubation. Cells that had migrated to the underside of the filters were fixed and photographed. Graph presents the invasion rate as the mean percentage of control. Error bars represent the mean ± SD of three independent experiments and * denotes *p* < 0.05 (Student’s *t*-test) between the two indicated groups. (**B**) Western blotting of indicated EMT marker proteins after two days of drug treatment in Caki-2 cells. Whole-cell extracts were immunoblotted with indicated antibodies. (**C**) The mRNA levels of EMT marker genes were evaluated after 24 h of drug treatment in Caki-2 cells. Error bars represent the mean ± SD of three independent experiments and * denotes *p* < 0.05 (Student’s *t*-test) between the two indicated groups.

As AR regulates major EMT-related genes including CDH1 and VIM, we expected that EMT markers would be affected by co-treatment with PG and enzalutamide. Cell infiltration decreased after enzalutamide or PG treatment alone, but co-treatment induced a significant and more dramatic decrease (Figure 5A). The mesenchymal markers N-cadherin, vimentin, and vascular endothelial growth factor (VEGF) also decreased upon either enzalutamide or PG treatment alone, but the reduction was again more pronounced in co-treated cells (Figure 5B). Although we failed to detect E-cadherin protein signals in this experimental condition, the mRNA level of CDH1 increased after co-treatment (Figure 5C). The mRNA level of CDH2 and VIM also decreased further after PG co-treatment (Figure 5C). These data show the additive effect of enzalutamide and PG on changes in the cell mobility of kidney cancer.

### 2.6. LSD1 is Essential for Kidney Cancer Growth in Xenograft Model

**Figure 6:**
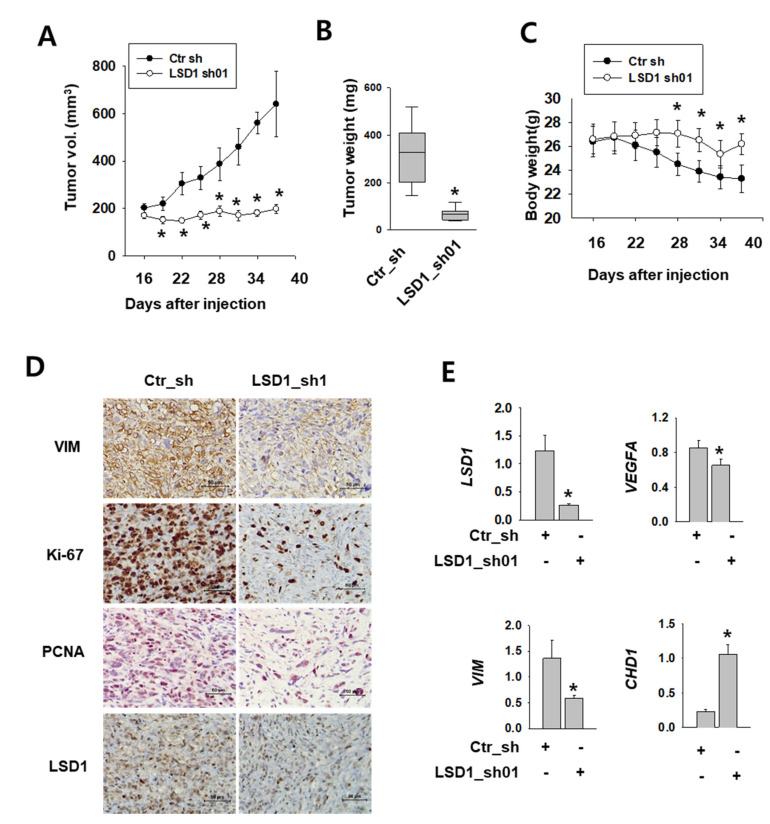
LSD1 repression attenuates tumor growth in human kidney cancer xenograft model. (**A**) Tumor size measured after injection of each indicated cell line into non-obese diabetic (NOD) severe combined immunodeficient (SCID) gamma (NSG) mice. Error bars represent the mean ± SEM (*n* = 6) of each group. * denotes *p* < 0.05 (Student’s *t*-test) versus the ctr_sh group. (**B**) Relationship between tumor weights and LSD1 expression in the human kidney xenograft model. Tumors were excised and examined at the end of the experiments. Representative graph is shown as vertical box plots, and tumor weights are presented as mean (g) ± SEM (*n* = 6). * denotes *p* < 0.05 (Student’s *t*-test) versus the ctr_sh group. (**C**) Mouse body weight was measured at indicated days after cancer cell injection. Error bars represent the mean ± SEM (*n* = 6) of each group. * denotes *p* < 0.05 (Student’s *t*-test) versus the ctr_sh group. (**D**) Immunohistochemical images of indicated antibodies in paraffin-embedded sections derived from the xenograft tumors. Scale bar, 50µm. (**E**) Quantitative PCR was performed for each gene using the mRNAs from each excised tumor. Error bars represent the mean ± SEM (*n* = 6) of each group. * denotes *p* < 0.05 (Student’s *t*-test) versus the ctr_sh group.

To test the role of LSD1 in kidney tumor growth in vivo, we implanted Caki-2 human kidney cancer cells expressing control or LSD1 shRNA into the flank of non-obese diabetic (NOD) severe combined immunodeficient (SCID) gamma (NSG) mice. After injection, we measured the tumor growth twice a week. The tumors expressing control shRNA were visible at 16 days after injection and grew constantly (Figure 6A). However, tumors expressing LSD1 shRNA did not grow during the same period of time. A significant difference was also evident in the tumor weight (Figure 6B). During tumor growth, the body weight decreased in mice with tumors expressing control shRNA, but not in mice bearing LSD1 shRNA-expressing tumors (Figure 6C). Next, we performed immunostaining of various cell-proliferating markers, such as Ki-67 and proliferating cell nuclear antigen (PCNA), which indicate rapid cell growth. LSD1 shRNA-expressing tumors showed reduced immunoreactivity for Ki-67 and PCNA, as well as for the mesenchymal marker vimentin (Figure 6D). Furthermore, we analysed mRNA levels of EMT markers in the extracted tumors and found that the epithelial marker CHD1 was up-regulated, whereas mesenchymal markers vascular endothelial growth factor A (VEGFA) and VIM were down-regulated (Figure 6E). These data confirmed the role of LSD1 in kidney cancer growth and metastasis in vivo.

## 3. Discussion

Although its role has been studied extensively in prostate cancer, growing evidence suggests AR plays a role in other cancers, including colon, breast, and bladder. In kidney cancer, however, the expression of AR is negatively correlated with cancer development [10,29]. Therefore, the role of AR in kidney cancer development received less attention. However, the fact that most AR proteins in normal kidney tissues are in the cytoplasmic fraction raised the possibility that AR amounts may not be directly linked with its actual function in kidney cancer. In fact, cellular localization and interaction with other co-factors may affect AR activity in kidney cancer. Moreover, several reports showed that AR has functions in kidney cancer progression [13,14,30]. Additionally, sunitinib induces AR phosphorylation in RCC, and AR inhibition by enzalutamide rescues sunitinib resistance in vivo [31]. Taken together, cellular localization and the regulation of AR by co-factors may play a more important role than its expression in kidney cancer development. 

In this study, we showed that LSD1 regulated AR activity in kidney cancer cells and demonstrated that pargyline, used as a LSD1 inhibitor, reduced the kidney cancer cell proliferation rate and metastatic ability. Monoamine oxidase (MAO) inhibitors, including pargyline, have been used to affect the proliferation and apoptosis of various cancer cells including breast [32,33], neuroblastoma [34], and prostate [26,35]; however, they have not yet been used for kidney cancer. Although several mechanisms related to the anti-cancer effect of MAO inhibitors have been demonstrated, the precise molecular mechanism was not clear. In our study, we specified AR as the target of LSD1 inhibitors in kidney cancer cells. We therefore designed a co-treatment strategy, using pargyline with AR inhibitor enzalutamide, which showed an additive effect on reducing kidney cancer cell proliferation and migration. Enzalutamide is a well-established treatment option for patients with metastatic castration-resistant prostate cancer (mCRPC). The known mode of action of enzalutamide on AR consists of three different stages, including inhibition of androgen binding to AR, inhibition of nuclear translocation of activated AR, and abrogation of AR binding to DNA [36]. Our data suggest, however, that the epigenetic regulation by PG on AR activity is distinct from the mode of action of enzalutamide. As numerous phase one and two clinical trials are assessing the addition of enzalutamide to current treatment regimens [37], co-treatment of PG with enzalutamide might be a good treatment option for enzalutamide-resistant cancer patients. As PG is already FDA-approved for human use, the clinical trials could be initiated more quickly than for newly developed drugs. 

In addition to AR, LSD1 also regulates other major transcription factors, including estrogen receptor (ER), c myeloblastosis (cMyb), GATA3, and NF-кB [38,39,40,41]. Among these, ER and GATA3 regulation is directly associated with breast cancer, and NF-кB regulation is also thought to influence the cell growth of various cancers, in addition to its main role in the control of immune processes. Therefore, the use of an LSD1 inhibitor in our experiment might also have affected the regulation of these transcription factors. As we only focused on AR activity and the regulation of its downstream genes, it might be interesting to study the effect of LSD1 inhibitors on the activity of other transcription factors, and co-treatment with their inhibitors in kidney cancer. For the purpose of investigating AR function with minimal confounding variables, we exclusively used the Caki-2 cell line, which expresses a high level of AR. Therefore, our results may not be applicable to all kidney cancer cell lines or all patients. However, our data suggest that targeting LSD1 and AR may be a good treatment option for kidney cancer patients screened for high-level AR expression.

In summary, we showed the epigenetic regulatory function of LSD1 on AR activity in kidney cancer development and migration, and the possible use of LSD1 inhibitors in kidney cancer therapeutics.

## 4. Materials and Methods 

### 4.1. Materials

Minimum essential media (MEM), Dulbecco′s modified eagle′s-medium (DMEM), trypsin, antibiotics, TRIzol and Lipofectamine 2000 were purchased from Invitrogen (Carlsbad, CA, USA). Foetal bovine serum (FBS) and culture media were obtained from Hyclone Laboratories Inc. (South Logan, UT, USA). Pargyline and other chemicals were purchased from Sigma-Aldrich (St. Louis, MO, USA). The antibodies used were as follows: anti-LSD1 antibody was purchased from ThermoFisher Scientific (Walldorf, Germany). Antibodies against AR (sc-816), VEGF (sc-7269), IGF1R (sc-712), and β-actin (sc-47778) were purchased from SantaCruz Biotechnology (Santa Cruz, CA, USA), and antibodies against cleaved PARP (#5625), cleaved caspase-9 (#9505), E-cadherin (E-Cad) (#3195), N-cadherin (N-Cad) (#13116), H3K9-2Me (#4658), and MYC (#5605) were from Cell Signaling (Danvers, MA, USA). Antibodies against vimentin (ab92547), PCNA (ab29), and Ki-67 (ab66155) were from Abcam (Cambridge, England).

### 4.2. Cell Lines, Cloning, and Virus Production

The Caki-2 and 293T cell lines were purchased from the Korean cell line bank (Seoul, Korea) in 2011. Cell lines were passaged no more than 15 times and authenticated by short tandem repeat analysis (Korean cell line bank) in 2018 (293T) and 2020 (Caki-2). We tested mycoplasma contamination using the e-MycoTM mycoplasma PCR detection kit (iNtRON Biotechnology, SungNam, Korea), and was negative in all cell lines. Caki-2 cells were cultured in MEM and 293T cells in DMEM medium, both supplemented with 10% FBS, at 37 °C in 5% CO_2_. For making shRNA-expressing vectors, the empty pLKO.1-puro lentiviral vector was purchased from Sigma-Aldrich, and annealed oligonucleotides for control shRNA or LSD1 shRNAs (sequences are listed in Supplemental Table S1) were inserted into the vector using the AgeI and EcoRI restriction enzymes. The detailed method for lentivirus packaging is as described [42].

### 4.3. RNA Isolation and RT-qPCR

For extracting total cellular RNA, we used the TRIzol reagent (Ambion, Austin, TX, USA) according to the manufacturer’s instructions. For cDNA synthesis 1 µg of total RNA was used, using the TOPscriptTM reverse transcription kit from Enzynomics (Daejeon, Korea). Real-time PCR was performed using the EvaGreen qPCR Master Mix (Applied Biological Materials Inc., Richmond, BC, Canada) and ABI 7500 Real-Time PCR System (Applied Biosystems, Waltham, MA, USA). Ribosomal RNA 18S was used as an internal control. The primer sequences used for RT-qPCR are listed in Supplemental Table S2.

### 4.4. Western Blotting of Cultured Cells

Cells (5 × 10^6^) were lysed in 1 mL radioimmunoprecipitation assay (RIPA) buffer (50 mM tris(hydroxymethyl)aminomethane-HCl (pH 7.2), 150 mM sodium chloride, 0.5% NP-40, 1% Triton X-100, and 1% sodium deoxycholate) containing 0.5 mM dithiothreitol and a protease inhibitor cocktail (Sigma-Aldrich). Protein extracts were quantitated with the bicinchoninic acid assay method, and separated on sodium dodecyl sulphate (SDS)-polyacrylamide gels, and Western blotting was performed as described [42].

### 4.5. Flow Cytometry for Analysis of Apoptosis and Cell Viability Assay

Flow cytometry was employed to analyse apoptosis using propidium iodide (PI) and an annexin V-fluorescein isothiocyanate (FITC) staining kit (BD Biosciences, San Jose, CA, USA). After harvesting, cells in binding buffer (1 × 10^6^ cells/mL) were incubated with PI and annexin V-FITC for 15 min at 25 °C in the dark. The cells were analysed with BD FACSCalibur cytometer (BD Biosciences). For the cell viability assay, 2000 to 3000 cells were dispensed in 100 μl culture medium in a 96-well plate and cultured for the indicated times. For the cell viability analysis, EZ-Cytox cell viability kit (Daeil-Lab, Korea) solution (10 μl) was added to each well of the plate. After being incubated for 1 h at 37 °C, the optical density (OD)_450_ was measured using a microplate reader (PerkinElmer, Waltham, MA, USA). 

### 4.6. Colony Formation Assay and Cell Invasion Assay

For the colony formation assay, each cell was counted, and 1000 cells were plated in 6-well plates. Cells were cultured for 14 days and stained with 0.1% crystal violet (Sigma-Aldrich) solution. The resulting plates were photographed and the number of colonies comprising of more than 50 individual cells was counted. For the invasion assay, cells (5 × 10^4^/well) were plated in the upper chambers of transwells with Matrigel-coated polycarbonate membranes (Corning, Big Flats, NY, USA). Media with 10% FBS was added into the lower chambers as a chemoattractant for cell migration. After 48 h, the migrated cells in lower chambers were fixed using 10% ethanol (Sigma-Aldrich). After cells were stained with the 0.01% crystal violet solution, migrated cells were randomly counted in five different microscopic fields at 20 × magnification.

### 4.7. Chromatin Immunoprecipitation (ChIP) Assays

The ChIP assay was performed as previously described [42]. In brief, 1 × 10^7^ cells were cross-linked with 1% formaldehyde for 10 min at room temperature, then quenched with glycine solution (final concentration, 125 mM). After lysis, the extracts were sonicated until the DNA fragments became < 500 base pairs. Each indicated antibody was added to chromatin complexes and incubated overnight at 4 °C. Protein A/G-sepharose beads were added and incubated for 2 more hours. After washing with saline, DNA-protein complexes were eluted with elution buffer (1% SDS, 0.1 M sodium bicarbonate (NaHCO_3_)) and eluted DNAs were incubated overnight at 65 °C to reverse the cross-links. The MEGA quick-spinTM DNA purification kit (Intron, Seoul, Korea) was used for purification. The purified DNAs were PCR quantified on a StepOneTM real-time PCR system, using the EvaGreen qPCR Master Mix (Applied Biological Materials Inc.). The PCR primer sequences used for the ChIP PCR are listed in Supplemental Table S3.

### 4.8. Animal Study

All animal experiments were performed in accordance with Seoul National University Hospital institutional guidelines under institutional animal care and use committee protocol no.16-0056-S1A8. NOD SCID gamma (NSG) mice were purchased from Jackson Laboratory (Bar Harbor, ME, USA), and maintained under specific pathogen-free (SPF) conditions. We injected 1 × 10^7^ human Caki-2 cells expressing the indicated shRNA into the right flank of six-week-old male NSG mice. For injection, cells were suspended with 100 µl of 50 % Matrigel (BD, NJ, USA) in complete media. Tumor growth was monitored externally using vernier calipers for up to 38 days after cell injection. Tumors were dissected and weighed on the final day. The tumors were divided into two parts and either fixed in neutral buffered formalin for immunohistochemistry (IHC) or frozen at −80 °C for RNA extraction.

### 4.9. Immunohistochemical Staining and Analysis

Slides from xenograft tumors were deparaffinized and stained with indicated antibodies. Then, photographs were taken under a Leica microscope (Wetzlar, Germany). 

### 4.10. Statistical Analyses

All data were analysed using Microsoft Excel software (version 2016, Microsoft, Redmond, WA, USA), unless otherwise stated. Continuous variables were analysed using Student’s *t*-test if the data were normally distributed. All statistical tests were two-sided. Differences were considered significant when *p* values were < 0.05.

## Supplementary Materials

Supplementary materials can be found at https://www.mdpi.com/1422-0067/21/17/6089/s1.

## Abbreviations

AR	androgen receptor
ChIP	chromatin immunoprecipitation
DHT	dihydrotestosterone
E-CAD	E-cadherin
EMT	epithelial-mesenchymal transition
EZH2	Enhancer of Zeste Homolog 2
IGF1R	insulin-like growth factor 1 receptor
KDM	lysine demethylase
KLK	kallikrein related peptidase
LSD1	lysine-specific histone demethylase 1
N-CAD	N-cadherin
PCAF	P300/CBP-associated factor
VEGF	vascular endothelial growth factor
VIM	vimentin
