# Supplementary material for: Histone Demethylase LSD1 Regulates Kidney Cancer Progression by Modulating Androgen Receptor Activity
Source: Int J Mol Sci. 2020 Aug 24;21(17):6089. doi: 10.3390/ijms21176089 (PMC7503698; doi:10.3390/ijms21176089)
Supplement: Supplementary file 1 [file ijms-21-06089-s001.pdf]

Histone demethylase LSD1 regulates kidney cancer progression by modulating androgen receptor activity

Kyoung-Hwa Lee[1], Byung-Chan Kim[1], Seung-Hwan Jeong[2], Chang Wook Jeong[1], Ja Hyeon Ku[1], Cheol Kwak[1,3,] * Hyeon Hoe Kim[1, 3,] *

[1] Department of Urology, Seoul National University Hospital, Seoul 03080, Korea; Lee12042@snu.ac.kr, dalkyal12@gmail.com, drboss@gmail.com, randyku@hanmail.net, hhkim@snu.ac.k

[2] Graduate School of Medical Science and Engineering, Korea Advanced Institute of Science and Technology (KAIST), Daejeon 34052, Korea; 11shjeong@gmail.com

[3] Department of Urology, Seoul National University College of Medicine, Seoul 03080, Korea

* Correspondence: mdrafael@snu.ac.kr; Tel: +822-2072-0817 (C.K.); hhkim@snu.ac.kr; Tel: +822-2072-2425 (H.H.K.)

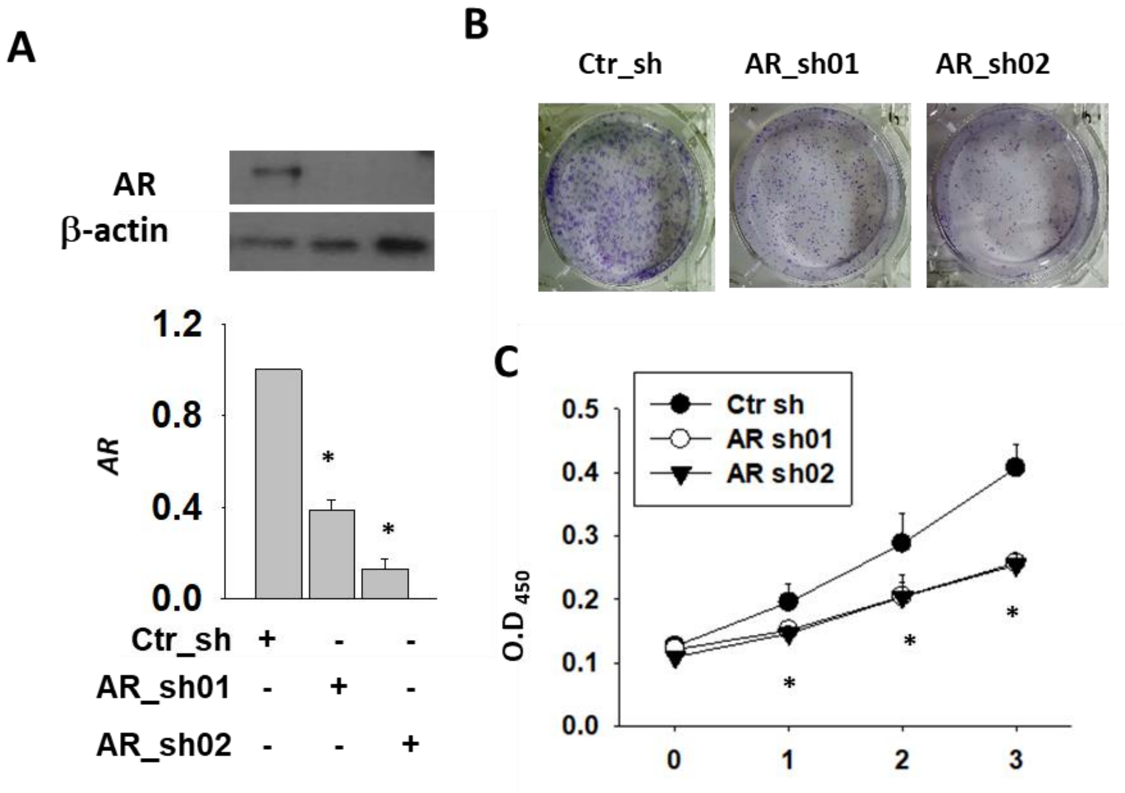

Supplemental Figure S1. (**A**) AR shRNA expression was confirmed by RT-PCR and western blotting in caki-2 cells. Bars represents the means ± SDs of three independent experiments, and * denotes P < 0.05 (student t-test) versus the control group (ctr_sh). (**B**) Crystal violet staining for colonies from same numbers of indicated shRNA-expressing Caki-2 cells (**C**) The time dependent viability changes of control shRNA and AR shRNA expressing caki-2 cells were measured using EZ-Cytox solution. Bars represents the means ± SDs of three independent experiments, and * denotes P < 0.05 (student t-test) versus the ctr_sh group.

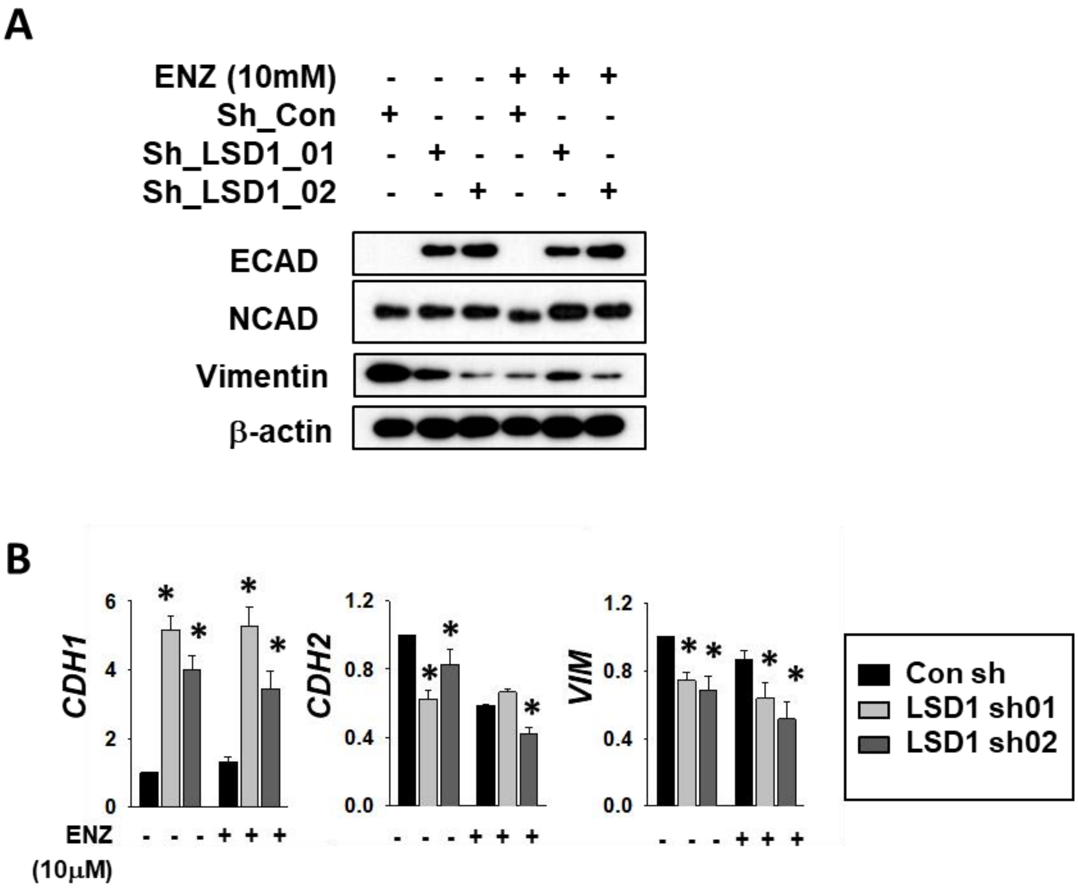

Supplemental Figure S2. LSD1 knock-down reduced cell mobility and EMT related gene expression in kidney cancer cell line. (**A**) Western blotting of indicated EMT markers after 24 hours treatment with enzalutamide (ENZ) on control or LSD1 shRNA expressing cells. (**B**) Quantitative PCR was used to examine the transcriptional level of the EMT marker genes in Caki-2 cells expressing control or LSD1 shRNA after 2 days of enzalutamide treatment. Error bars represent the mean ± SD of three independent experiments and * denotes P < 0.05 (student's t-test) versus ctr_sh group.

Supplemental Table S1. Oligonucleotide sequences shRNA lentiviral vector cloning

Upper_control_shRNA	CCGGCGTGATCTTCACCGACAAGATCTCGAGATCTTGTCGGTGAAGATCACGTTTTT
Bottom_control_shRNA	AATTAAAAACGTGATCTTCACCGACAAGATCTCGAGATCTTGTCGGTGAAGATCACG
upper_LSD1(h)_shRNA01	CCGGACCGGATGACTTCTCAAGAAGCTCGAGCTTCTTGAGAAGTCATCCGGTTTTTTT
bottom_LSD1(h)_shRNA01	AATTAAAAAAACCGGATGACTTCTCAAGAAGCTCGAGCTTCTTGAGAAGTCATCCGGT
upper_LSD1(h)_shRNA02	CCGGCACAAGGAAAGCTAGAAGAAACTCGAGTTTCTTCTAGCTTTCCTTGTGTTTTTT
bottom_LSD1(h)_shRNA02	AATTAAAAAACACAAGGAAAGCTAGAAGAAACTCGAGTTTCTTCTAGCTTTCCTTGTG

Supplement Table S2. Oligonucleotide sequences for RT

18S_rRNA_RT_Fwd	TTCGTATTGAGCCGCTAGA
18S_rRNA_RT_Rev	CTTTCGCTCTGGTCCGTCTT
hLSD1_RT_Fwd	CCTTCACCCCACCAAGAGAC
hLSD1_RT_Rev	AGACGTTGTTTGGCTGTTGC
hKLK3_RT_Fwd	CACCTGCTCGGGTGATTCTG
hKLK3_RT_Rev	CCACTTCCGGTAATGCACCA
hKLK2_RT_Fwd	GCTGCCCATTGCCTAAAGAAG
hKLK2_RT_Rev	TGGGAAGCTGTGGCTGACA
hTMPRSS2_RT_Fwd	GGACAGTGTGCACCTCAAAGA
hTMPRSS2_RT_Rev	TTGCTGCCCATGAACTTCC
hIGF1R_RT_Fwd	GGGCCATCAGGATTGAGAAA
hIGF1R_RT_Rev	CACAGGCCGTGTCGTTGTCA
hVEGF_RT_Fwd	TGCATTCACATTTGTTGTGC
hVEGF_RT_Rev	AGACCCTGGTGGACATCTTC
hAR_RT_Fwd	AGGATCGCTCGTCTCTGGTA
hAR_RT_Rev	GGAGCTCTGCACTCACTTCT
hMYC_RT_Fwd	TACAACACCCGAGCAAGGAC
hMYC_RT_Rev	TTCTCCTCCTCGTCGCAGTA
hCDH1_RT_Fwd	TCGGACCAAGGACAAGTACC
hCDH1_RT_Rev	ATCTTCACCTGCCGTTCAGT
hCDH2_RT_Fwd	GACAATGCCCCTCAAGTGTT
hCDH2_RT_Rev	CCATTAAGCCGAGTGATGGT
hVIM_RT_Fwd	GAGAACTTTGCCGTTGAAGC
hVIM_RT_Rev	GCTTTCTGTAGGTGGCAATC

Supplemental Table S3. Oligonucleotide sequences for ChIP

hKLK3 _ChIP_Fwd	GGGATCAGGGAGTCTCACAA
hKLK3 _ChIP_Rev	GCTAGCACTTGCTGTTCTGC
hKLK2_ChIP_Fwd	GCCTTCTCTGGCTTTGTTCC
hKLK2_ChIP_Rev	GCACTTGCTGTTCCACACAT
hTMPRSS2_ChIP_Fwd	TGGTCCTGGATGATAAAAAAAGTTT
hTMPRSS2_ChIP_Rev	GACATACGCCCCACAACAGA
